# Effects of BNN27, a novel C17-spiroepoxy steroid derivative, on experimental retinal detachment-induced photoreceptor cell death

**DOI:** 10.1038/s41598-018-28633-1

**Published:** 2018-07-13

**Authors:** Pavlina Tsoka, Hidetaka Matsumoto, Daniel E. Maidana, Keiko Kataoka, Irene Naoumidi, Achille Gravanis, Demetrios G. Vavvas, Miltiadis K. Tsilimbaris

**Affiliations:** 1 Laboratory of Optics and Vision, University of Crete Medical School, Heraklion, Crete Greece; 2 Department of Pharmacology, University of Crete Medical School, Heraklion, Crete Greece; 3 Institute of Molecular Biology and Biotechnology, Foundation for Research and Technology-Hellas, Heraklion, Crete Greece; 4 Angiogenesis Laboratory, Retina Service, Department of Ophthalmology, Massachusetts Eye and Ear Infirmary, Harvard Medical School, Boston, Massachusetts USA

## Abstract

Retinal detachment (RD) leads to photoreceptor cell death secondary to the physical separation of the retina from the underlying retinal pigment epithelium. Intensifying photoreceptor survival in the detached retina could be remarkably favorable for many retinopathies in which RD can be seen. BNN27, a blood-brain barrier (BBB)-permeable, C17-spiroepoxy derivative of dehydroepiandrosterone (DHEA) has shown promising neuroprotective activity through interaction with nerve growth factor receptors, TrkA and p75^NTR^. Here, we administered BNN27 systemically in a murine model of RD. TUNEL^+^ photoreceptors were significantly decreased 24 hours post injury after a single administration of 200 mg/kg BNN27. Furthermore, BNN27 increased inflammatory cell infiltration, as well as, two markers of gliosis 24 hours post RD. However, single or multiple doses of BNN27 were not able to protect the overall survival of photoreceptors 7 days post injury. Additionally, BNN27 did not induce the activation/phosphorylation of TrkA^Y490^ in the detached retina although the mRNA levels of the receptor were increased in the photoreceptors post injury. Together, these findings, do not demonstrate neuroprotective activity of BNN27 in experimentally-induced RD. Further studies are needed in order to elucidate the paradox/contradiction of these results and the mechanism of action of BNN27 in this model of photoreceptor cell damage.

## Introduction

During retinal detachment (RD), photoreceptors are physically separated from the retinal pigment epithelium (RPE), the underlying supporting nourishing tissue of the retina. This separation activates a signaling cascade that culminates in photoreceptor cell death mediated by significant cross talk between apoptosis, regulated necrosis and other cell death pathways^1–5^. Following retinal detachment, macrophages and microglia infiltrate into the subretinal space^4–7^, while Müller cells and astrocytes proliferate, migrate and hypertrophy within the retina^8,9^. The photoreceptor cell loss that ensues, results in suboptimal visual outcome for many patients. Mechanisms that can enhance photoreceptor survival could be particularly beneficial for many retinopathies that involve photoreceptor separation from the RPE.

Dehydroepiandrosterone (DHEA), the most abundant steroid in the plasma, is a well-characterized neurosteroid^10,11^ and a notable neuroprotective molecule due to its ability to prevent neuronal cell death on various experimental neurodegenerative models both *in vivo* and *in vitro*^12–17^, partially through interaction with the neurotrophin family receptors; tyrosine kinase receptor (TrkA, TrkB, TrkC) and/or p75^NTR^^18–21^. However, DHEA is an intermediate in the biosynthesis of androgens and estrogens and thus treatment with this steroid can be problematic due to potential endocrine side effects^22–25^. For this reason, effort has been made to develop analogues that will retain the anti-apoptotic properties while inhibiting their ability to convert to estrogens or androgens. BNN27, is a novel synthetic C17-spiroepoxy [(R)-3β, 21-dihydroxy-17R, 20-epoxy-5-pregnene] steroid derivative of DHEA with such properties^26^. BNN27 retains DHEA’s neurotrophic activity by selective binding to TrkA receptor and subsequent induction of its phosphorylation and downstream survival signaling in primary cultures of NGF-dependent primary sympathetic neurons^27^. BNN27 can rapidly enter the mouse central nervous system (CNS)^28^ and can significantly diminish caspase-3 mediated cell death in the dorsal root ganglia of NGF null mice embryos^27^. Furthermore, BNN27 was able to protect mature oligodendrocytes in an animal model of multiple sclerosis (MS)^29^ and reverse the diabetes-induced loss of immunoreactivity of retinal amacrine cells and ganglion cell axons’ markers in an experimental model of diabetic retinopathy (DR)^30^. Finally, BNN27 was neuroprotective in a co-culture of mouse motor neurons with human astrocytes from amyotrophic lateral sclerosis (ALS) patients, however, it did not improve several clinical characteristics of the SOD1 mouse model of the disease^31^.

Based on the above, in the present study, we investigated whether systemically administered BNN27 can protect photoreceptors from cell death in the murine model of experimental retinal detachment and how BNN27 administration can affect the detached retina.

## Results

### BNN27 reduces TUNEL^+^ photoreceptors after RD

**Figure 1:**
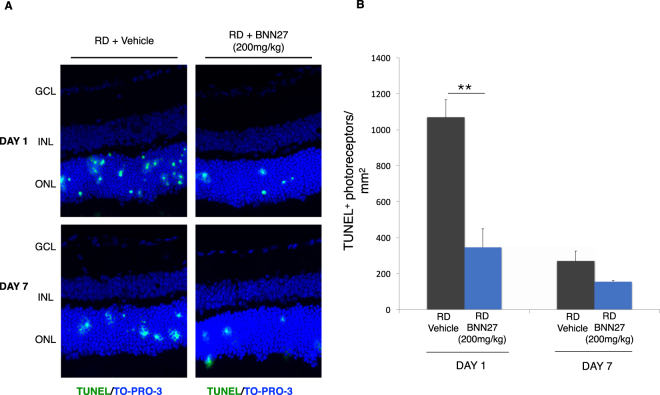
Effect of BNN27 on RD-induced cell death. (**A**) TUNEL (green) and TO-PRO-3 (blue) staining of retinal sections from untreated (vehicle) and BNN27-treated eyes, 24 hours and seven days post RD. (**B**) 24 hours post RD, BNN27-treated group showed significantly lower numbers of TUNEL^+^ photoreceptors (cells/mm^2^), *n* = 15, ^**^P < 0.01. On the contrary, 7 days post RD, BNN27 treatment did not reach a statistically significant level of reduction of TUNEL^+^ photoreceptors (cells/mm^2^), *n* = 6–7. Scale bar: 100 μm. The graph shows mean ± SEM. RD, Retinal Detachment, ONL, Outer Nuclear Layer, INL, Inner Nuclear Layer, GCL, Ganglion Cell Layer.

To determine the potential neuroprotective effect of BNN27 on the photoreceptors after experimental retinal detachment (RD), we examined the RD-induced cell death in the outer nuclear layer (ONL) by TUNEL assay. Photoreceptor cell death peaks at 24 hours post RD and wanes by day 7^4,32,33^. A single intraperitoneal injection of BNN27 (200 mg/kg), 60 minutes post RD, decreased TUNEL^+^ cells by 65% on day 1 (RD + Vehicle: 1068 ± 99 cells/mm^2^, RD + BNN27: 346 ± 102 cells/mm^2^, ^**^P < 0.01, *n* = 15) but did not result in statistically significant difference on day 7, *n* = 6–7 (Fig. 1A,B).

### BNN27 induces macrophage/microglia infiltration following RD

**Figure 2:**
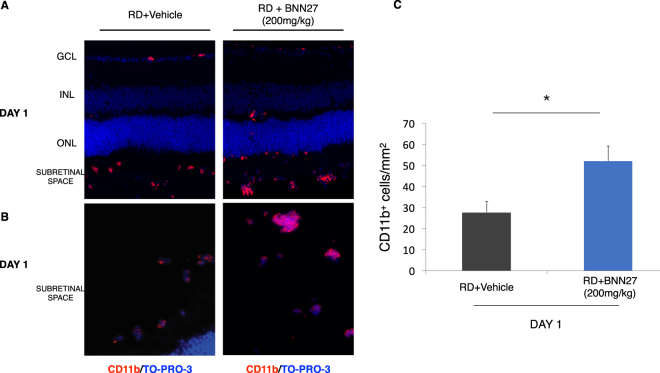
Effect of BNN27 on RD-induced macrophage/microglia infiltration. (**A**) CD11b (red) and TO-PRO-3 (blue) staining 24 hours post RD, Scale bar: 500 μm. (**B**) CD11b (red) and TO-PRO-3 (blue) staining. Large aggregates of CD11b^+^ cells in the subretinal space of the BNN27-treated eyes, scale bar: 100 μm. (**C**) Infiltration of CD11b^+^ cells was significantly higher in the group which received BNN27 treatment 24 hours after RD, *n* = 12, ^*^P < 0.05. The graph shows mean ± SEM. RD, Retinal Detachment, ONL, Outer Nuclear Layer, INL, Inner Nuclear Layer, GCL, Ganglion Cell Layer.

Retinal detachment promotes an accumulation of CD11b^+^ macrophages and activated microglia in the retina and more specifically in the subretinal space^4–7,32,33^. We previously reported that in our model the peak of the infiltration of CD11b^+^ cells into the subretinal space coincides with the peak of photoreceptor cell death 24 hours after RD^4^. Thus, we examined the effect of BNN27 on macrophage/microglia infiltration by detecting the macrophage/microglial marker CD11b by immunofluorescence 24 hours post RD. BNN27-treated group displayed a significant increase of the CD11b^+^ cells compared to vehicle-treated (RD + BNN27: 52 ± 7 cells/mm^2^ vs. RD + Vehicle: 27 ± 5 cells/mm^2^, ^*^P < 0.05, *n* = 12, Fig. 2A and C). In addition to individual CD11b^+^ cells, clusters of CD11b^+^ cells were also found in both groups. Again, BNN27-treated animals had more and larger clusters (Fig. 2B).

### BNN27 increases RD-induced gliosis

**Figure 3:**
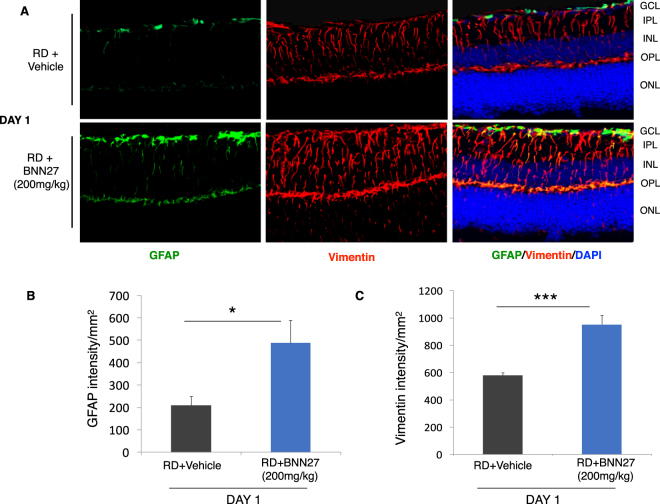
Effect of BNN27 on RD-induced gliosis. (**A**) Representative images of Vimentin (red), GFAP (green) and DAPI (blue) staining 24 hours post RD. (**B** and **C**). GFAP and vimentin intensity were significantly higher in the BNN27-treated group 24 hours post RD (^*^P < 0.05 and ^*^P < 0.001 respectively), *n* = 9. Scale bar: 100 μm. The graphs show mean ± SEM. RD, Retinal Detachment, ONL, Outer Nuclear Layer, OPL, Outer Plexiform Layer, INL, Inner Nuclear Layer, IPL, Inner Plexiform Layer, GCL, Ganglion Cell Layer.

RD triggers the activation and proliferation of glial cells, a response known as reactive gliosis^34^. Reactive gliosis is characterized by morphological alterations in astrocytes and Müller cells and by increased expression of glial fibrillary acidic protein (GFAP) and vimentin^8,9,34,35^. To investigate the action of BNN27 on RD-induced gliosis, retinal sections were stained with anti-GFAP and anti-vimentin antibodies. Both GFAP and vimentin intensity/mm^2^ were significantly increased in the BNN27-treated group 24 hours post detachment (RD + Vehicle: 209 ± 38 mean gray value/mm^2^, RD + BNN27: 487 ± 102 mean gray value/mm^2^, *n* = 9, ^*^P < 0.05 for GFAP and RD + Vehicle: 578 ± 20 mean gray value/mm^2^, RD + BNN27: 952 ± 67 mean gray value/mm^2^, *n* = 9, ^***^P < 0.001 for vimentin, Fig. 3A,B and C).

### BNN27 does not induce TrkA phosphorylation, although the mRNA levels of the receptor are elevated in the detached photoreceptors

**Figure 4:**
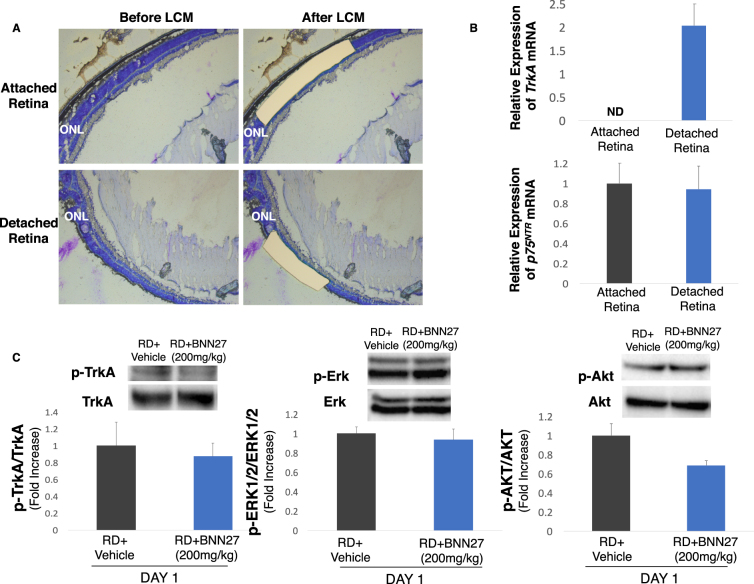
Expression of TrkA and p75^NTR^ in photoreceptors and effect of BNN27 on TrkA phosphorylation and downstream signaling following RD. (**A**) Representative pictures of retinal sections before and after cutting the ONL with LCM from attached and detached retina. Nuclei were stained with toluidine blue. (**B**) *TrkA* and *p75*^*NTR*^ mRNA expression in the ONL following isolation of the photoreceptors’ nuclei with LCM. *TrkA* mRNA levels were not detected in the attached retina while they were significantly elevated in the detached, *n* = 4–5. On the contrary, *p75*^*NTR*^ mRNA levels were not altered before and after injury, *n* = 4–5. (**C**) Western blotting images and densitometry analysis of phosphorylated TrkA, total TrkA, phosphorylated Erk, total Erk, phosphorylated Akt and total Akt of detached retinas between untreated and BNN27-treated eyes. BNN27 did not further induce phosphorylation of TrkA, Erk or Akt, *n* = 4. Scale bar: 100 μm. The graphs show mean ± SEM. RD, Retinal Detachment, ONL, Outer Nuclear Layer, LCM, Laser Capture Microdissection, ND, Not Detectable.

NGF has been extensively studied in retinal degenerations^4,36–43^, however, the expression of its receptors, TrkA and p75^NTR^ in healthy^19,38,39,44–48^ and degenerated^38,42,44–46,48^ photoreceptors has not been fully elucidated. To clarify this point, we examined the mRNA levels of *TrkA* and *p75*^*NTR*^ in the outer nuclear layer (ONL) in both healthy and detached retina by laser capture microdissection (LCM) (Fig. 4A). *TrkA* mRNA was not detected in the ONL before injury while it was robustly increased 24 hours post RD (Fig. 4B, *n* = 4–5). On the contrary, there was no significant change in the mRNA levels of *p75*^*NTR*^ before and after injury (Fig. 4B, *n* = 4–5), indicating that at least in the photoreceptors p75^NTR^ does not play an instrumental role following RD. BNN27 selectively binds to TrkA receptor leading to its phosphorylation and promoting neuroprotection in a TrkA-dependent manner^27^. We have previously shown that phosphorylation, thus activation of TrkA, is elevated following experimental RD^4^. To assess if BNN27 can further upregulate TrkA activation, we examined the phosphorylation of the receptor on Y^490^ residue and the downstream signaling which leads to neuronal survival and differentiation in BNN27-treated and untreated detached retinas. Interestingly, phosphorylation of TrkA was not significantly increased in the BNN27-treated group and consequently neither was phosphorylation of Akt or Erk (phosphorylated-to-total ratio, Fig. 4C, *n* = 4).

### BNN27 does not protect the outer nuclear layer (ONL) thickness

**Figure 5:**
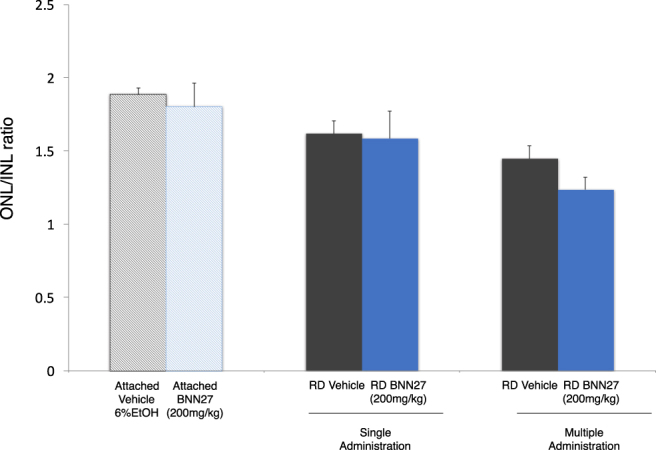
Effect of BNN27 on outer nuclear layer (ONL) thickness. Ratio of ONL/INL in the attached and the detached retina following RD. At day 7, single or multiple (daily administration, 7 injections total) doses of BNN27 were not able to protect the overall thickness of the ONL of the detached retina, *n* = 6–7. The graph shows mean ± SEM. RD, Retinal Detachment, ONL, Outer Nuclear Layer, INL, Inner Nuclear Layer.

Given the opposing effects of BNN27 on TUNEL positivity, inflammatory/gliotic markers and lack of activation of TrkA downstream signaling following RD, we wanted to evaluate what is its overall impact on survival of photoreceptor nuclei (ONL) at day 7 post injury. As depicted in Fig. 1, a single systemic administration of BNN27 led to a significant reduction in TUNEL^+^ cells at day 1 post RD but did not prevent the loss of photoreceptors (ONL thickness) by day 7, *n* = 6–7 (Fig. 5). To examine if more frequent administration of BNN27 could lead to rescue of ONL, the experiment was repeated with seven daily administrations of BNN27, *n* = 6–7. However, even the frequent dosing did not lead to rescue of the ONL (Fig. 5).

## Discussion

Separation of photoreceptors from the underlying/supporting RPE results in photoreceptor cell loss and visual dysfunction and can be seen in many disorders such as rhegmatogenous RD (RRD), age-related macular degeneration (AMD)^49^, diabetic retinopathy (DR)^50^ and retinopathy of prematurity (ROP)^51^. In the case of RRD, surgical re-apposition of the retina to the RPE is a well-established therapeutic approach, however, visual acuity is not always restored^52^. Understanding the cellular mechanisms of photoreceptor cell loss will aid in identifying potential therapeutic targets for effective neuroprotection and improved visual function.

DHEA has received significant attention for its neuroprotective activity^12–21^. Lately, the interest has been intensified because of the discovery that DHEA binds to and activates all tyrosine kinase (Trk) receptors, as well as, the pan-neurotrophin receptor p75^NTR^^18–21^. The ability of DHEA to activate neurotrophin receptors further expands its potential for neuroprotection. TrkA receptor is preferentially activated by nerve growth factor (NGF) and is associated with neuronal survival and differentiation^53^. We have previously shown that *NGF* mRNA levels are elevated following experimental RD^4^. Additionally, exogenous administration of NGF reduced RD-induced photoreceptor cell death^38^ and protected the retinal neurons in various animal models, including retinitis pigmentosa (RP)^36,42^, retinal ischemia-reperfusion injury^43^ and DR^41^. Nonetheless, administration of NGF was not always protective in retinal degeneration^37,39,40^. Furthermore, DHEA was able to rescue TrkA^+^ sensory neurons in NGF null embryos^18^, while inhibition of TrkA reversed the neuroprotective effect of DHEA and/or NGF in the inner retina in a model of AMPA-induced retinal excitotoxicity^19^. However, given the considerable clinical limitations of DHEA, due to its effects on the endocrine axis and its conversion to multiple androgen and estrogen metabolites^22–25^, several groups have synthesized a handful number of novel DHEA derivatives to mitigate this problem/effect^26,54,55^. Among them, to the best of our knowledge, only the spiro-analogs of DHEA (BNNs) have been tested and reported to have neuroprotective activity^26,27,29,30,56,57^. BNN27 can protect PC12 cells from serum deprivation-induced apoptosis^26,27^, can reduce TUNEL^+^ cell death in superior cervical ganglia following NGF deprivation^27^ and can also diminish caspase-3 mediated cell death in dorsal root ganglia of NGF null embryos^27^, in serum-deprived PC12 cells^27^ and in cuprizone^29^- and diabetes^30^-induced apoptosis. Ιn addition, and in contrast to polypeptidic neurotrophins that cannot cross the blood-brain barrier (BBB)^58^, the small lipophilic BNN27 can cross the BBB and can be detected in the mouse brain 30 minutes after intraperitoneal administration^28^. Taken together, all these findings suggest that BNN27 could be a potent neuroprotective agent in acute retinal injury and photoreceptor degeneration.

In the present study, we administered BNN27 for the first time in an *in vivo* model of retinal photoreceptor degeneration. BNN27 given systemically can be detected by HPLC chromatography in the rat retina two hours after intraperitoneal injection with a peak at four hours post administration^59,60^. We showed that a single dose of BNN27 given 60 minutes after RD injury significantly reduces TUNEL-positive photoreceptor cell death at 24 hours (the peak of cell death in this model^4,32,33^) but not at 7 days.

Because cell death is associated with inflammation, and because activators of TrkA have reported immunomodulatory effects^21,61^, we examined the effects of BNN27 in the inflammatory response seen after RD. Systemic administration of BNN27 significantly increased the number of infiltrating macrophages/microglia in the subretinal space and to a lesser extent in the retina. Not only the number of the CD11b^+^ cells was significantly higher in the treated group but also their distribution was altered with noticeable increase in the presence of large aggregates of CD11b^+^ cells. NGF activation of TrkA/p75^NTR^ can increase microglial migration^62^ and can induce macrophage-mediated tumor necrosis factor (TNF)-α production^63^, interleukin-1β (IL-1β) secretion and inflammasome activation^64,65^. Our data indicate that BNN27, probably by mimicking NGF action or cooperating with it, enhances the immune response of RD by increasing the numbers of infiltrating macrophages and microglia. Our results are, however, in contrast with two recent studies showing that BNN27 administration can reduce Iba-1^+^ microglia and pro-inflammatory cytokines in the cuprizone-induced experimental multiple sclerosis (MS)^29^ or in the streptozotocin-induced experimental diabetic retinopathy (DR)^30^. At the same time, BNN27 increases anti-inflammatory cytokine, interleukin-10 and -4 (IL-10 and IL-4 respectively), levels in diabetic retinas^30^. Although the infiltrating macrophages/microglia between the BNN27-treated and the untreated detached retinas did not show any significant differences in the expression of inducible nitric oxide synthase (iNOS) or arginase-1 (*n* = 4–5), two out of many markers for M1 and M2 macrophages respectively^66^, the subtype of the inflammatory cells in our study remains to be further investigated. In addition, it must be ascertained if the observed increase in the infiltrating cells following BNN27 administration is beneficial or not given that M2 macrophages can induce anti-inflammatory cytokine production and secretion such as IL-10 and IL-4^66^. Furthermore, although CD11b and Iba-1 are both expressed by macrophage and microglia populations, each marker alone cannot discriminate resident microglia from infiltrating macrophages^67–69^ so perhaps BNN27 has an opposite effect on these two populations. Further research is needed in order to characterize the effect of BNN27 on those two distinct types of inflammatory cells.

Retinal detachment injury results in reactive gliosis and is characterized by the activation of Müller cells and astrocytes. Upon activation of these cells, there is an increased production of the intermediate filament proteins, GFAP and vimentin and also characteristic alterations in their morphology. Activation of GFAP expression by Müller cells is also seen in proliferation and dedifferentiation. NGF has been found to modulate retinal gliosis and decrease GFAP levels in different models of retinal degeneration or injury^38,48,70^. On the other hand, NGF acts as a mitogenic signal for Müller cells and thus increases Müller cells’ proliferation and dedifferentiation^48,71–73^, hence the effect of NGF treatment in the injured retina might be detrimental. In our study, intraperitoneal administration of BNN27 significantly increased the production of the above-mentioned proteins and further altered the morphology of the GFAP^+^ glial cells. On the contrary, BNN27 was able to reduce the GFAP-mediated astrogliosis in experimental diabetic retinopathy^30^, in which diabetes affects the inner retina, the Müller cells and the retinal ganglion cells (RGCs). Indeed, photoreceptors, Müller cells and RGCs have different patterns of NGF/pro-NGF and/or TrkA expression during degeneration^74^. Furthermore, it is important to note that overactivation of Müller Glia (MG) can also be a potential therapeutic target due to their ability of reprogramming and thus becoming reparative towards injury^75,76^. Future studies are necessary in order to elucidate if BNN27-induced overactivation of retinal glial cells is beneficial or detrimental to the retina, secondary to the primary injury.

Expression of TrkA and p75^NTR^ has been extensively studied in the healthy rodent retina as well as in different models of inherited retinal degenerations and retinal injuries^4,19,38,39,42,44–48,74^. However, thus far, it is uncertain if TrkA is expressed in healthy photoreceptors^19,38,39,44,47,74^. Although previous studies have shown immunoreactivity of TrkA in the outer nuclear layer (ONL)^38,47^, the specificity of the antibody was questioned^19,39,47,48,74^. In our study, we showed that mRNA levels of *TrkA* are not detectable in healthy photoreceptors, isolated by laser capture microdissection (LCM), in agreement to a single previous study which used the same method in rats^44^. Also, we showed for the first time, that 24 hours post RD, mRNA levels of *TrkA* are significantly elevated in the photoreceptors, in line with a previous study in which TrkA was detected by immunohistochemistry in the detached retinas^38^. On the contrary, in a different model of experimental retinal degeneration, only the mRNA levels of *TrkC* were altered in the photoreceptors after intense light exposure and no difference was observed in the levels of *TrkA* or *TrkB*^44^. In contrast to *TrkA*, *p75*^*NTR*^ mRNA levels were detectable in the attached healthy retina, in accordance to previous studies that have verified the expression of p75^NTR^ in healthy photoreceptors by various methods^45,46^. However, there was no significant upregulation following RD injury. Likewise, *p75*^*NTR*^ mRNA levels were not altered in photoreceptors after light injury as was detected by LCM^44^, although, in another study p75^NTR^ was significantly elevated in photoreceptors in the same type of injury as was detected by *in situ* hybridization and immunostaining^46^. Furthermore, elevated levels of p75^NTR^ in photoreceptors were also detected by electron microscopy in an experimental model of retinal dystrophy^45^. The heterogeneity in the expression of Trk and p75^NTR^ in photoreceptors following injury and/or degeneration implicates that various detrimental stimuli to photoreceptors result in different modulation of Trk and/or p75^NTR^ receptors. Nonetheless, p75^NTR^ might be upregulated during experimental RD in other retinal cell types, and thus its expression in the total detached retina has to be further investigated.

BNN27 selectively binds to TrkA receptor^27,29,30^, induces its phosphorylation^27,30,77,78^ and upregulates the expression of phospho-Erk^27,77,78^ and phospho-Akt^27^, while in the absence of TrkA receptor, BNN27 binds to and activates the p75^NTR^ receptor and consequently protects the murine cerebellar granule neurons from serum deprivation-induced apoptosis^56^. Given the elevated mRNA levels of *TrkA* in detached photoreceptors and the reported induction of TrkA phosphorylation following RD^4^, we examined if BNN27 can further activate the TrkA receptor and the downstream neuroprotective signaling. Surprisingly, BNN27 was not able to significantly induce the phosphorylation of TrkA, Akt or Erk proteins. A possible explanation is that BNN27 does only slightly upregulate TrkA phosphorylation in this type of injury and primarily in different cells (e.g. Müller cells) than photoreceptors. In that case, given the low ratio of any retinal cell population compare to photoreceptors, western blotting might lack sensitivity and single cell western blotting or other techniques might be required to detect such slight differences if any. The different way of how each retinal cell population reacts to degeneration/trauma should also be taken into account. BNN27 significantly induced TrkA and Erk phosphorylation in experimental DR^30,77,78^, a chronic metabolic disease of the inner retina. Our data indicate that BNN27 might have a different mechanism of action in photoreceptors and/or in acute trauma in CNS through interaction with other DHEA’s receptors.

Given the opposing effects of BNN27 on TUNEL^+^ cells, inflammation/gliosis and activation of TrkA signaling, we wanted to see its overall effect on preservation of photoreceptors in the outer nuclear layer (ONL) by day 7. Despite the reduction in TUNEL^+^ cells at 24 hours (the peak of cell death in our model^4,32,33^), we were not able to detect any differences in the ONL thickness between the BNN27-treated and the untreated group seven days post RD either after a single or after multiple administrations of BNN27 (200 mg/kg). To evaluate further if a different dosing regimen is needed for optimal effects of BNN27, we administered BNN27 at three more doses (10 mg/kg, 50 mg/kg and 100 mg/kg) daily for seven days. However, we were not able to see any differences between the treated and the untreated eyes (*n* = 6–9 for each of the three groups). These results could suggest that there is an overall shift in the cell death kinetics and/or that additional inhibitors of cell death pathways are needed. Indeed, BNN27 has only been reported to reduce markers primarily associated with apoptosis (caspase-3, TUNEL)^26,27,29,30,56^, while it has been extensively documented that RD-induced cell death is mediated by a perplexed crosstalk between various cell death pathways^1–5^. On the other hand, the lack of BNN27-induced TrkA phosphorylation might be responsible for the lack of the overall protection. Similar to our results, another study has shown that although BNN27 rescues mouse motor neurons co-cultured with human astrocytes from patients with ALS with the SOD1 mutation, it failed to show an overall effect on neuropathological markers in an *in vivo* model of ALS in mice^31^. On the contrary, systemic administration of BNN27 was able to protect the brain nitric oxide synthase (bNOS)- and tyrosine hydroxylase (TH)- expressing amacrine cells as well as preserve the ganglion cell axons in a rat model of DR^30^. Nonetheless, in the same study BNN27 was not able to reduce the TUNEL^+^ cells in two different paradigms of administration^30^. Furthermore, in a different study, BNN27 reduced cuprizone-induced apoptosis in mature oligodendrocytes but did not prevent demyelination in the same challenge^29^. Taken all together, BNN27 seems to have a very divergent effect on different models of CNS neurodegeneration. Each deleterious stimulus activates distinct cell signaling combinations and perhaps RD has a very different nature given the acute ischemic trauma to the retina compare to the chronic metabolic diabetic retinopathy or the inflammatory demyelinating multiple sclerosis. Furthermore, important differences in the CNS between mice and rats have been reported several times in the past including different patterns of neurogenesis^79^, response upon stimuli/trauma^80,81^ and pharmacology^82^. Likely, differences between the two species could be another possible explanation for the contradictory results of BNN’s potential neuroprotective activity in different models of retinal neurodegeneration along with the nature of the disease.

The paradox of decreased TUNEL^+^ cells with the increased macrophage infiltration and gliosis markers, concurrently with the lack of TrkA activation, appears to be quite complex and could explain why overall ONL thickness was unaltered despite a drastic reduction in observed TUNEL^+^ cell death. Furthermore, neuroinflammation and gliosis are not necessarily neurotoxic; they include both neuroprotective and neurotoxic signals. The shift between these two signals is still unclear and the mechanism of action of BNN27 on the inflammatory cells and the glial cells of the retina remains to be elucidated. In summary, our study was not able to conclude if BNN27 has overall neuroprotective activity in the RD model. More extensive studies with different dosing and/or models are needed to assess the potential therapeutic role of this novel microneurotrophin in diseases like RD affecting the outer retinal layers.

## Materials and Methods

### Animals

All animal experiments followed the guidelines of the Association for Research in Vision and Ophthalmology (ARVO) Statement for the Use of Animals in Ophthalmic and Vision Research and were approved by the Animal Care Committee of Massachusetts Eye and Ear Infirmary. C57BL/6 male mice (7–10 weeks) were purchased from Charles River Laboratories (Wilmington, MA, USA) and had free access to food and water in an air-conditioned room with a 12-h light/12-h dark cycle.

### Experimental Model of Retinal Detachment

Our previously reported modified experimental approach for retinal detachment^83^ was followed for all the experiments. In brief, mice were anesthetized with an intraperitoneal injection of ketamine (60 mg/kg, Ketaved; Ketamine HCL 100 mg, Vedco Inc., Saint Joseph, MO, USA) and xylazine (6 mg/kg, Anased Injection 20 mg; Lloyd Inc., Shenandoah, IA, USA) and proparacaine drops (0.5% Proparacaine Hydrochloride Ophthalmic Solution; Sandoz Inc., Princeton, NJ, USA) were also applied for topical anesthesia. Pupils were dilated with a topical applied mixture of phenylephrine (5%) and tropicamide (0.5%) (Massachusetts Eye and Ear Infirmary Pharmacy, Boston, MA, USA). Next, a conjunctival incision was made over the temporal aspect of the eye and a sclerotomy was created approximately 3–4 mm to the limbus. Subsequently, a corneal paracentesis was made to lower intraocular pressure. Finally, a 10-µl syringe (NanoFil; WPI, Sarasota, FL, USA or Hamilton, 701RN SYR, #7635-01; Hamilton Company, Reno, NV, USA) with a 33- or a 34-gauge needle (Hamilton Custom Needles: Length: 10.00 mm/Point Style: 4/Angle: 20, #7803-05; Hamilton Company, Reno, NV, USA or 34g beveled NanoFil needle, #NF34BV-2; WPI, Sarasota, FL, USA) was inserted into the subretinal space and 4 µl of 1% sodium hyaluronate (Provisc; Alcon, Fort Worth, TX, USA) were injected gently to detach the retina from the underlying RPE. Approximately 60% of the temporal-nasal neurosensory retina was detached. At the end of the procedure, cyanoacrylate surgical glue (Webglue; Patterson Companies, Mendota Heights, MN, USA) was applied on the scleral wound to prevent leaking and keep the conjunctiva attached to the original position. Special care was given to avoid hitting the lens. Eyes with subretinal hemorrhage or cataract were excluded from the analysis. Antibiotic ointment (Bacitracin Zinc Ointment; Fougera Pharmaceuticals Inc, Melville, NY, USA) was applied topically as a last step to prevent microbial infection.

### BNN27 Injections

BNN27 was obtained from Bionature E.A. Ltd (Nicosia, Cyprus). The stock solution (150 mg/ml) was prepared by diluting 60 mg of BNN27 in 400 μl of absolute ethanol at 57–60 °C until the solution was clear. BNN27 was administered intraperitoneally. Animals received one injection of BNN27 (200 mg/kg, diluted in 6% absolute ethanol in water) or vehicle (6% absolute ethanol in water) one hour post RD or received a total of seven injections of BNN27 (200 mg/kg, diluted in 6% absolute ethanol in water) or vehicle (6% absolute ethanol in water) starting one hour post RD and then administered once daily.

### TUNEL (TdT-dUTP terminal nick-end labeling) assay

Mice were euthanized 24 hours or 7 days post RD and eyes were enucleated, embedded in O.C.T. compound (Tissue Tek; Sakura Finetek, Torrance, CA, USA) and fresh-frozen at −80 °C. Serial sections were cut in the sagittal plane at 10 µm-thickness on a cryostat (Leica CM1850; Leica Biosystems, Buffalo Grove, IL, USA) and fixed in 4% paraformaldehyde (PFA), followed by TUNEL assay analysis according to the manufacturer’s protocol, omitting post-fixation (ApopTag Fluorescein *In Situ* Apoptosis Detection Kit #S7110; MilliporeSigma, Burlington, MA, USA). Finally, sections were counterstained with TO-PRO-3 Iodide (642/661) (Life Technologies #T3605; Thermo Fisher Scientific, Waltham, MA) and mounted with Fluoromount-G (SouthernBiotech, Birmingham, AL, USA). Images were taken with an upright AXIO Imager.M2 Zeiss fluorescence microscope and were analyzed using Zeiss ZEN software (Carl Zeiss Inc., Thornwood, NY, USA).

### Immunofluorescence

Animals were euthanized 24 hours post RD, eyes were enucleated and serial sections were taken as described above. Subsequently, sections were fixed in 4% PFA, blocked with 5% bovine serum albumin (BSA) and incubated overnight at 4 °C with anti-Vimentin (1:200, Millipore #AB5733; MilliporeSigma, Burlington, MA, USA) and anti-Glial Fibrillary Acidic Protein (GFAP) antibodies (1:200, Dako #Z0334; Agilent Technologies, Santa Clara, CA, USA) or fixed in acetone, blocked in 5% milk and incubated overnight at 4 °C with anti-CD11b antibody (1:50, BD Pharmingen #550282; BD Biosciences, San Jose, CA, USA). Following the primary antibody incubation, the sections were stained with goat anti-chicken 647, goat anti-rabbit 488 and goat anti-rat 488 respectively (1:500, Alexa-Fluor 647 goat anti-chicken #A-21449; Alexa-Fluor 488 goat anti-rabbit #A-11034; Alexa-Fluor 488 goat anti-rat #A-11006; respectively, Molecular Probes, Thermo Fisher Scientific, Waltham, MA, USA). Finally, sections were counterstained with TO-PRO-3 Iodide (642/661) (Life Technologies #T3605; Thermo Fisher Scientific, Waltham, MA) or DAPI and mounted as described above. Images were taken with an upright AXIO Imager.M2 Zeiss fluorescence microscope and were analyzed using Zeiss ZEN software (Carl Zeiss Inc., Thornwood, NY, USA).

### Laser Capture Microdissection (LCM)

Mice were euthanized 24 hours post RD, eyes were enucleated, embedded in O.C.T. compound (Tissue Tek; Sakura Finetek, Torrance, CA, USA) and fresh-frozen at −80 °C. Eyes were then cut in the sagittal plane at 20 µm-thickness on a cryostat (Leica CM1850; Leica Biosystems, Buffalo Grove, IL, USA) and serial sections were collected on polyethylene terephthalate-membrane (PET) frame slides (PET FrameSlide #0010; steel frames, RNase-free, material number #11505190, Leica Microsystems, Wetzlar, Germany). Sections were fixed in 75% ethanol (30 seconds), washed with nuclease-free water (30 seconds), stained with 0.02% toluidine blue solution for 20 seconds and washed again as described above. Finally, sections were dehydrated with 75%, 95% and 100% ethanol (30, 30 and 2 × 30 seconds respectively). LCM was performed with the Leica LMD7000 system and LMD application version 7.5 (Leica Microsystems, Wetzlar, Germany). Photoreceptors’ layer was cut by laser and collected into 0.5 ml tubes containing RNA*later* stabilization solution (Invitrogen, Thermo Fisher Scientific, Waltham, MA, USA).

### RNA extraction and RT-PCR

RNA extraction was achieved with RNeasy plus micro kit (Qiagen, Germantown, MD, USA) according to manufacturer’s protocol. cDNA was synthesized with SuperScript III Reverse Transcriptase and Oligo(dT)_20_ Primer following manufacturer’s instructions (Invitrogen, Thermo Fisher Scientific, Waltham, MA, USA). Real-time PCR was carried out by StepOnePlus Real-Time PCR System (Applied Biosystems, Thermo Fisher Scientific, Foster City, CA, USA). Reactions were performed with TaqMan Fast Universal PCR Master Mix, no AmpErase UNG (Thermo Fisher Scientific, Waltham, MA, USA) and TaqMan primers [*18s rRNA*: Mm03928990_g1; *TrkA*: Mm01219406_m1; *p75*^*NTR*^: Mm00446296_m1 (TaqMan Gene Expression Assay (FAM), Thermo Fisher Scientific, Waltham, MA, USA)]. The relative quantity of mRNA expression was calculated by ΔΔ Ct method normalized to *18s rRNA* as endogenous control.

### Western Blotting

Animals were euthanized 24 hours post RD, retinas were dissected and immediately immersed in ice-cold lysis buffer containing 20 mM NaHEPES, 20 mM KCl, 20 mM NaF, 20 mM glycerophosphate, 2 mM sodium pyrophosphate, 1 mM sodium orthovanadate, 1% Triton-X-100 and a cocktail of protease inhibitors (cOmplete, Mini; Roche #11836170001, MilliporeSigma, Burlington, MA, USA). Total retinal lysates (each lysate contained two retinas) were sonicated (20% amplitude, 5 seconds, 2 times at 4 °C) and centrifuged (17,000 × g, 20 minutes at 4 °C). Supernatants were electrophoresed onto 4–12% Bis-Tris polyacrylamide gels (NuPage; Invitrogen #NP0321, Thermo Fisher Scientific, Waltham, MA, USA) and proteins were transferred on a 0.45 μm PVDF membrane (Immobilon-P; Millipore #IPVH00010, MilliporeSigma, Burlington, MA, USA). After blocking with 5% BSA in 1% Triton-X-100 in Tris-buffered saline (TBS) the membranes were incubated overnight at 4 °C with primary antibodies [TrkA (1:1500, #ab76291; Abcam, Cambridge, MA, USA) phospho-TrkA (Tyr490), p44/42 MAPK (Erk1/2), phospho-p44/42 MAPK (Erk1/2) (Thr202/Tyr204), Akt, phospho-Akt (Ser473) and β-actin (1:1000, #9141; #4695; #4370; #4691; #4060; #4970; respectively, Cell Signaling, Danvers, MA, USA)]. Following primary antibody incubation, the membranes were incubated with HRP-labeled secondary antibodies. Bands were detected by a chemiluminescent reagent (Amersham ECL Select Western Blotting Detection Reagent #RPN2235; GE Healthcare Life Sciences, Chicago, IL, USA) and images were taken with ChemiDoc MP (Bio-Rad Laboratories, Hercules, CA, USA).

### Evaluation of Outer Nuclear Layer (ONL)/Inner Nuclear Layer (INL) Ratio

Mice were euthanized 7 days post RD, eyes were enucleated and serial sections were taken as described above. Following fixation in 4% PFA, sections were stained with Hematoxylin solution, Gill No. 2, counterstained with 0.25% Eosin Y solution and mounted with VectaMount Permanent Mounting Medium (Vector Laboratories, Burlingame, CA, USA). Images were taken and analyzed as described previously.

### Quantification Analysis

For the quantification of TUNEL^+^ cells each section was examined under a 20×/0.8 lens (Zeiss PLAN-APOCHROMAT, Carl Zeiss Inc., Thornwood, NY, USA). To evaluate the TUNEL^+^ cell density, the total number of TUNEL^+^ cells in the ONL was counted and the area (of the ONL) was measured by Image J software (developed by Wayne Rasband, National Institutes of Health, Bethesda, MD). We previously reported that the center of RD had less variability of TUNEL^+^ cells^4^, therefore sections were collected around 1000 µm from the injection site. Shrunk part of the retina was excluded from the counting because mechanical stress can accelerate photoreceptor cell death. The average of two parts of the retina, one from either side of the detached retina, was calculated as the representative TUNEL^+^ photoreceptor cell density per section.

For the quantification of CD11b^+^ cells each section was examined under a 10×/0.3 lens (Zeiss EC-PLAN NEOFLUAR, Carl Zeiss Inc., Thornwood, NY, USA). To calculate the CD11b^+^ cell density, the total number of CD11b^+^ cells in the retina and in the subretinal space were counted and the whole area (retina and subretinal space) was measured by Image J software (developed by Wayne Rasband, National Institutes of Health, Bethesda, MD).

For the calculation of GFAP and vimentin intensity, each section was examined under a 20×/0.8 lens (Zeiss PLAN-APOCHROMAT, Carl Zeiss Inc., Thornwood, NY, USA). To assess the intensity per area, the gray mean value and the area (ganglion cell layer and inner plexiform layer) were calculated and measured by Image J software (developed by Wayne Rasband, National Institutes of Health, Bethesda, MD).

For the evaluation of the ONL/INL ratio, the outer nuclear layer (ONL) and the inner nuclear layer (INL) thickness of the retina were measured by ImageJ software (developed by Wayne Rasband, National Institutes of Health, Bethesda, MD) at 2 points of each section and ONL/INL ratio was calculated.

The average of three consecutive sections (with a step of 150 μm) was estimated as the representative measurement of each eye for all the above-mentioned quantifications.

### Statistical analysis

Statistical analysis was performed with GraphPad Prism 7 (La Jolla, CA, USA) using Student’s *t*-test (Figs 1, 2, 3 and 4) or one-way ANOVA followed by post analysis with Tukey HSD test (Fig. 5). Data were presented as the mean value ± SEM. The significance level was set at *P* < 0.05 (* in figures), *P* < 0.01 (** in figures) and *P* < 0.001 (*** in figures).

### Data availability

The datasets generated and/or analyzed during the current study are available from the first and/or the corresponding authors on reasonable request.
